# Structure and regulatory mechanisms of food‐derived peptides in inflammatory bowel disease: A review

**DOI:** 10.1002/fsn3.4228

**Published:** 2024-06-18

**Authors:** Wenpei Qiu, Zhicheng Wang, Qirui Liu, Qiwei Du, Xiaoqun Zeng, Zhen Wu, Daodong Pan, Xiaohong Zhang, Maolin Tu

**Affiliations:** ^1^ State Key Laboratory for Managing Biotic and Chemical Threats to the Quality and Safety of Agro‐Products Ningbo University Ningbo Zhejiang China; ^2^ Zhejiang‐Malaysia Joint Research Laboratory for Agricultural Product Processing and Nutrition, College of Food Science and Engineering Ningbo University Ningbo China; ^3^ School of Medicine Ningbo University Ningbo China

**Keywords:** food‐derived bioactive peptide, inflammatory bowel disease, peptide structure, regulatory mechanism

## Abstract

The number of patients with inflammatory bowel disease (IBD) is increasing worldwide. Since IBD is a chronic disease that seriously affects patients' life quality, preventing and alleviating IBD with natural and less side effect substances has become a research hotspot. Food‐derived bioactive peptides have been an attractive research focus due to their high efficiency and low toxicity. This paper comprehensively summarizes food‐derived peptides with intestinal health effects, focusing on peptide sequences with IBD‐regulatory effects and emphasizing the effects of their structure and physicochemical properties such as peptide length, amino acid composition, and net charge on their function. We also analyzed its regulatory mechanisms, mainly in 5 aspects: modulating the intestinal microbiota, decreasing intestinal epithelial permeability, increasing antioxidant ability, regulating the expression of inflammatory cytokines, and targeting signaling pathways. This review will help establish novel, efficient screening methods for IBD‐regulatory peptides and contribute to further research and discovery of them.

## Graphical abstract

Food‐derived peptides with huge potential to alleviate IBD. The identified peptides against IBD were listed and their structure properties were analyzed. IBD‐regulatory peptides act their functions through 5 mechanisms.

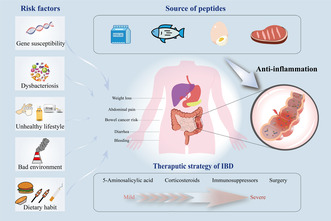

## 1 INTRODUCTION

**FIGURE 1:**
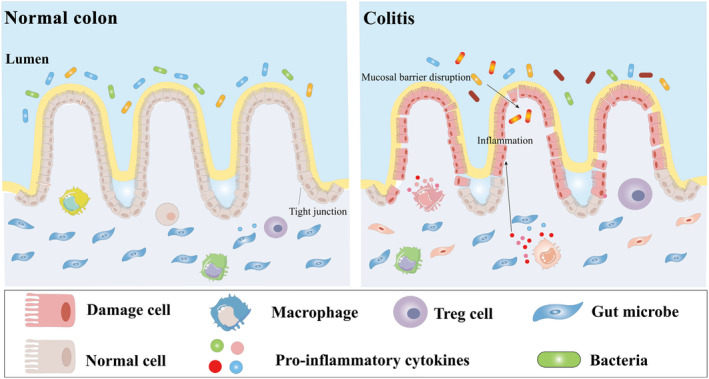
Comparison of normal and colitis intestinal tissues. In normal homeostasis, colonic tissue is in a healthy condition and works well, while in colitis, colon tissue is damaged and normal function will be affected.

Inflammatory bowel disease (IBD) is a group of non‐specific chronic gastrointestinal inflammatory diseases of unknown cause, including ulcerative colitis (UC) and Crohn's disease (CD). Thereinto, UC is a disease with long duration, occurring mostly in the mucosa or submucosa of the colon and rectum, accompanied by histologic changes (as shown in Figure 1), whereas CD commonly occurs in the distal ileum, colon, and crissum and may involve the entire digestive tract in severe conditions, which can be clearly distinguished from UC (Guo et al., 2022; Kobayashi et al., 2020). IBD has a complex etiology with a long course and a high recurrence rate, while the incidence of UC has seen a gradual increase for the past few years. Accordingly, it is worth further exploration and research to find more effective treatments.

Although the cause of UC is still uncertain, the following aspects may be potential factors (Ungaro et al., 2017): genetic factors, dysbacteriosis, environmental implications (e.g., air pollution), and unhealthy lifestyle (e.g., eating fried, frozen, or chilled food regularly, smoking and drug abuse) (Kobayashi et al., 2020; Mahid et al., 2006). Immunocompromise may be a direct cause of IBD, and gut microbes greatly influence the host's immune system (Schirmer et al., 2019). In addition, some researchers have found that the oral microbiota may also be involved in the pathogenesis of colitis. Oral bacteria can influence intestinal microecological balance through blood‐borne or enteric pathways, and participate in the development of IBD; in turn, IBD also affects the composition of the oral microbiome and leads to oral lesions (Han et al., 2022). Therefore, it is crucial to maintain host microecological balance. Statistics from various data show that the number of cancer patients developed from colitis is countless year by year with an increasing incidence rate. Therefore, it becomes increasingly important to intervene in this disease.

Currently, drugs for the treatment of UC include 5‐aminosalicylic acid, corticosteroids, and immunosuppressors. 5‐Aminosalicylic acid is widely used to cure patients with mild‐to‐moderate colitis. For patients who have failed or are intolerant to 5‐aminosalicylic acid treatment, oral/rectal corticosteroid therapy or in combination with 5‐aminosalicylic acid is an alternative option. Immunosuppressive therapy is recommended for moderate to severe patients (Panés & Alfaro, 2017). Corticosteroids such as budesonide (Abdalla & Herfarth, 2016) enhance local efficacy while limiting systemic bioavailability. In addition, the use of budesonide in UC is limited to rectal preparations, and oral administration of budesonide will lead to a decrease in its systemic bioavailability during metabolizing. Immunosuppressants, such as tumor necrosis factor (TNF) antagonists, and interleukin blockers, can relieve the symptoms of colitis by blocking inflammatory factors in the body. The use of TNF antagonists is an important advance in the treatment of colitis. At present, tumor antagonists used in the treatment of UC include infliximab (Laharie et al., 2018), adalimumab (Panés et al., 2022), and golimumab (Perrig et al., 2022). Infliximab is the earliest biological agent used to relieve UC, and golimumab has been discovered and approved in recent years to relieve moderate‐to‐severe colitis symptoms (Flamant et al., 2017; Hyams et al., 2020). New drug‐like substances, such as Janus kinase inhibitors, regulators of sphingosine‐1‐phosphate receptors, and SMAD‐7 antisense oligonucleotides, are still under study (Panés & Alfaro, 2017). However, these chemical drugs have side effects to a certain extent, so increasing research on the treatment for colitis focuses on natural ingredients, such as polysaccharides (Lin et al., 2017; Tang et al., 2021), bioactive peptides (Xiang, Jiang, et al., 2021; Xiang, Zhou, et al., 2021), probiotics (Stange et al., 2021), natural saponins (Cheng et al., 2022), and plant extracts (Yang et al., 2023).

In recent years, food‐derived bioactive peptides have attracted extensive attention due to their high safety. Bioactive peptides are one of the most important active substances that play a significant role in human growth, disease, aging, and death, with almost no side effects. Besides general studies, some researchers have conducted clinical trials to verify the health effect of some proteins or active peptides on colitis. In human clinical trials, bovine colostrum showed potential as a novel therapy for left‐sided colitis with additional benefits over using mesalazine alone (Khan et al., 2002). A human pilot study showed that the addition of casein glycomacropeptide as a nutritional therapy to standard treatment was safe and accepted by patients with active distal UC, and its disease‐modifying effect was similar to that of the mesalazine dose escalation (Hvas et al., 2016). It follows that food‐derived substances hold great promising in the treatment of colitis.

Currently, several discussions about the effects of peptides on regulating colitis have appeared. In addition to exploring the activity and role of a few potential colitis regulatory peptides, it seems to focus more on anti‐inflammatory peptides (mainly based on in vitro experiments). However, in vitro experiments alone are not enough to fully understand the impact of these peptides on IBD regulation, and in vivo experiments are crucial for the active studies of IBD‐regulatory peptides. Here, we comprehensively illustrated the current research status of IBD‐regulatory peptides with verified activities based on animal and intestinal cell models and summarized their structure characteristics (e.g., peptide length, amino acid composition, and net charge) in order to relate their functional activity to their structure, and we proposed some speculations to advance the screening and identification of IBD‐regulatory peptides, hoping to contribute to the discovery of IBD regulatory peptides.

## 2 RESEARCH STATUS OF FOOD‐DERIVED COLITIS REGULATORY PEPTIDES

### 2.1 Source of colitis regulatory peptides

Plenty of studies have verified that plant, animal, microbe, and aquatic proteins are good sources of bioactive peptides. Numerous studies have shown that proteins or their hydrolysates have activity in alleviating or preventing colitis. Some studies have identified active peptide sequences. Here, we summarized the basic information of the reported ones, as shown in Table 1. Colitis regulatory peptides have already been identified in animal proteins (tuna, duck egg white, and preserved egg white), milk (casein and lactoferrin), plant‐based proteins (corn protein, walnut protein, and rice protein), etc. (Table 1).

**TABLE 1:** Food‐derived peptides with beneficial effects on colitis.

No.	Peptide sequence	Source	Preparation methods	Activity mechanisms	Experimental model	References
1	EATKCFQWQRNMRKVRGPPVSCIKR	Human lactoferrin	Chemical synthesis	Diminish the IL‐1β, IL‐6, and TNF‐α levels, increase macrophages' number and IL‐10, remission of a shortened colon, reduce the occult blood in the fecal and colon	DSS‐induced colitis in C57BL/6J mice (male, 8 weeks)	Håversen et al. (2003)
2	TK(C)FQWQRNMRKVRGPPVS(C)IKR					
3	LPF	Walnut protein	Chemical synthesis	Decrease the levels of TNF‐α and IL‐6, regulate the imbalance of intestinal flora, protect the intestinal barrier, and increase microbial diversity and the population of Treg cells in spleen	DSS‐induced colitis in BALB/c mice (male, 6–7 weeks old, 18–20 g)	Zhi et al. (2022)
4	SDIKHFPF	*Tricholoa matsutake* Singer	Chemical synthesis	Relieve pathological changes in tissues, increase TJ protein ZO‐1 and occludin, reduce IL6, IL‐1β, TNF‐α, MPO, and DAO production	DSS‐induced colitis in BALB/c mice (male, 6–8 weeks old, 20 ± 2 g)	Li, Ge, et al. (2021)
5	SDLKHFPF					
6	IPVA	Whey protein	Enzymolysis	Reduce the TNF‐α‐induced signaling pathways, ERK1/2, NF‐κB, p38 MAPK, JNK1/2, and IL‐8 expression	In vitro TNF‐α‐stimulated Caco‐2 cells	Oyama et al. (2017)
7	EAMAPK	Stracchino cheese	In vitro gastro‐intestinal digestion	Reduce ROS production, increase SOD expression, and activate Nrf2 antioxidant response	In vitro H_2_O_2_‐treated intestinal epithelial cell line (IEC‐6)	Pepe et al. (2016)
8	AVPYPQ					
9	PyroEY	Japanese rice wine (*sake*)	Fractionation	Alleviate colitis symptoms: weight loss and shortened colon length, increase the DAI score, and balance the intestinal microbiome	DSS‐induced colitis in C57BL/6 mice (male, 7‐week‐old)	Kiyono et al. (2016)
10	pyroENI					
11	pyroEL	Wheat gluten hydrolysate	Liquid phase peptide synthesis	Alleviate colitis symptoms: weight loss and shortened colon length, increase the DAI score, improve F/B of colon, and balance the intestinal microbiome	DSS‐induced colitis in C57BL/6 mice (male, 7‐week‐old)	Wada et al. (2013)
12	PVLGPVRGPFPLL	Fresh wheat germ and apple	Fermentation by *Lactobacillus*	In vitro: increase TJ protein expression (ZO‐1, Claudin‐1, Occludin), keep epithelial barrier integrity, and lower permeability; In vivo: alleviate colitis, reduce IL‐6 expression, activate ERK, and TOR signaling pathways	In vitro human colonic epithelial cells (NCM460) In vivo DSS‐induced UC in C57BL/6J mice (male, 8‐week‐old)	He et al. (2022)
13	CKYVCTCKMS	*Bubalus bubalis* Mozzarella Cheese	Solid phase peptide synthesis	Reduce the phosphorylation of IκB‐α and active NF‐κB expression	2,4,6‐Dinitrobenzenesulfonic acid (DNBS)‐induced colitis in adult ICR mice (Male, 25–30 g)	Tenore et al. (2019)
14	DEDTQAMPFR	Preserved duck egg white	Chemical synthesis	Inhibit the phosphorylation of NF‐κB and MAPK pathways, downregulate IL‐8, IL‐1β, IL‐6, TNF‐α, and IL‐12 expression and upregulate the IL‐10	DSS‐induced colitis in Balb/c mice (Female, 7‐week‐old)	Zhang et al. (2018)
15	MLGATSL					
16	SLSFASR					
17	MSYSAGF					
18	VPY	Soy	Soy hydrolysate	Downregulate the production of pro‐inflammatory cytokines expression of TNF‐α, IL‐6, IL‐1β, IFN‐γ, and IL‐17	DSS‐induced acute colitis in Balb/c mice (female, 18–20 g)	Kovacs‐Nolan et al. (2012)
19	KCRQWQSKIRRTNPIFCIRR	Porcine lactoferrin	Chemical synthesis	Prevent colon epithelium tissue impairment, increase the expression of ZO‐1, occludin, and claudin‐1 and histological evidence of inflammation, reduce permeability and apoptosis, and inhibit the levels of TNF‐α, IL‐6, and IFN‐γ	LPS‐induced intestinal damage in C57/BL6 mice (male, 6–8 weeks)	Zong et al. (2016)
20	IRW	Egg white protein transferrin	Chemical synthesis	Upregulate the antioxidant enzymes (SOD, GSH‐Px, and catalase) and improve their antioxidant activities. Increase intestinal microbial diversity and enhance the abundance of gut microbiota	DSS‐induced colitis in ICR mice (female, about 22.5 g)	Liu et al. (2018)
21	IQW	Egg white protein ovotransferrin		Up‐regulate the antioxidant enzymes (SOD, GSH‐Px, catalase) and improve their antioxidant activities. Increase intestinal microbial diversity and enhance the abundance of gut microbiota	DSS‐induced colitis in ICR mice (female, about 22.5 g)	Liu et al. (2018)
	IQW	Egg white protein transferrin	/	Strengthen the ability of anti‐inflammatory agents, promote the expression of SCFAs, increase probiotic abundance, and regulate the intestinal flora and metabolic changes	DSS‐induced colitis in C57BL/6J (ICR) mice (female, 6‐week‐old)	Chai et al. (2022)
22	GPA	Fish skin gelatin	Chemical synthesis	Increase the expression of Nur 77, inhibit the pro‐inflammatory NF‐κB signaling pathway, and repair the intestinal epithelial barrier	In vitro mouse intestinal epithelial cell line (MODE‐K). In vivo DSS‐induced colitis in C57BL/6 mice (male, 5‐week‐old)	Deng, Zheng, et al. (2020)
23	GPR	Fish skin gelatin	Chemical synthesis	Inhibit the pro‐inflammatory cytokine production of IL‐6, IL‐8, and IL‐12	In vitro LPS induced‐inflammation in IPEC‐J2 cells	Deng, Cui, et al. (2020)
24	GP(Hyp)					
25	L(Hyp)G					
26	(Hyp)P					
27	LNLYP	Chicken by‐product	Chemical synthesis and purification	Activate aryl hydrocarbon receptor (AhR) activation and inhibit the Src kinase to increase TJ protein levels	DSS‐induced colitis in C57BL/6 mice (male, 18–22 g)	Li et al. (2020)
28	LPLLR	Walnut	Chemical synthesis	Scavenge ROS and alleviate oxidative stress via activating Nrf2/Keap1 pathway, increase CAT and SOD expression, inhibit NLRP3 inflammasome activation, reduce IL‐18, IL‐1β, and TNF‐α release, and increase IL‐10	In vitro H_2_O_2_‐induced oxidative stress in Caco‐2 cells In vivo DSS‐induced colitis in C57BL/6 mice (male, 6‐week‐old)	Qi et al. (2023)
29	TPGAFF	Quinoa	Chemical synthesis	Inhibit NO release, reduce IL‐1β and TNF‐α, increase TJ proteins and COX‐2 expression, inhibit NF‐κB pathway, increase *Bacteroides*, *Blautia*, and *Lachnospiraceae*, and reduce intestinal permeability	In vitro LPS‐induced inflammation in raw 264.7 cells In vivo DSS‐induced colitis in C57BL/6j mice (male, 8‐week‐old)	Wang et al. (2024)
30	LLTRAGL	Rapana venosa	Chemical synthesis	Reduce the migration of macrophages to the intestine, enhance intestinal peristalsis, and improve intestinal inflammatory damage	2,4,6‐trinitrobenzene sulfonic acid (TNBS)‐induced colitis in zebrafish	Cao et al. (2024)
31	VSAAAA	Millet gliadin	Enzymolized by pepsin	Reduce MPO, IL‐6, TNF‐α, and IL‐1β, increase IL‐10 expression, and increase TJ protein levels (ZO‐1, occludin, claudin‐1, claudin‐3), inhibit NF‐κB pathway	DSS‐induced colitis in C57BL/6 mice (males, 6‐week‐old)	Hong et al. (2023)
32	SHTLP	Walnut	Chemical synthesis	Inhibit activation of the TLR4‐MAPK pathway, reduce harmful bacteria (*Helicobacter* and *Bacteroides*), increase beneficial bacteria abundance (*Candidatus_Saccharimonas*), inhibit TNF‐α, IL‐6, IL‐1β, and increase IL‐10 levels, and improve TJ protein expression	DSS‐induced colitis in C57BL/6 mice (males, 6‐week‐old)	Chen et al. (2023)
33	HYNLN					
34	LGTYP					
35	SSEDIKE	Amaranth protein	Chemical synthesis	Attenuate cell activation, inhibit CCL20 mRNA coding the expression and NF‐κB pathway	In vitro Caco‐2 and Caco‐2 transfected with a luciferase reporter	Fernandez‐Tome et al. (2019)
36	GLTSK	Bean	Chemical synthesis	Reduce DAI scores, decrease the rate of shortening of the colon, and the number of tumors in the distal region	AOM/DSS‐induced colitis in Balb/c mice	Luna‐Vital et al. (2017)
37	WFNNAGP	Tricholoma matsutake	Chemical synthesis	Ameliorate oxidative stress, scavenge hydroxyl and DPPH radicals, increase SOD, decrease MDA and MPO activity, promote occludin and ZO‐1, downregulate the IL‐1β, IL‐6, and TNF‐α expression and NF‐κB pathway, and inhibit the formation and activation of NLRP3 and caspase‐1	DSS‐induced colitis in BALB/c mice (6–8‐week‐old)	Li, Lv, et al. (2021)
38	APEPEPAF	Wheat germ‐derived peptide	Chemical synthesis	Promote the expression of TJ proteins (claudin‐1, ZO‐1, and occludin); decrease the abundance of *Bacteroides* and increase the abundance of *Dubosiella* and *Lachnospiraceae_UCG‐006*, reduce IL‐6, IL‐1β, TNF‐α, and MPO levels, and inhibit the PKCζ/NF‐κB pathway	DSS‐induced colitis in C57BL/6 mice (male, 6–8‐week‐old)	Wang et al. (2023)

### 2.2 Identification of colitis regulatory peptides

Although numerous peptides with regulatory effects on colitis have been identified, they represent only a small fraction of potential therapeutic agents, with the majority yet to be discovered. One of the important obstacles to finding more colitis regulatory peptides was that there was no efficient and economical method to screen the colitis regulatory peptides. Limited studies identified the colitis regulatory peptides through the following methods: evaluate the anti‐oxidant and/or anti‐inflammatory activity of peptides by cell experiments (Zhao et al., 2023), and then verify the colitis regulatory effects on animal models mainly containing DSS‐induced/TNBS‐induced colitis mouse models (Ghattamaneni et al., 2018). Researchers usually use Raw 264.7 models to validate the anti‐inflammatory effects of peptides, detecting the mRNA and protein expression levels of various cell cytokines (Liu et al., 2019). Caco‐2 cells are applied to evaluate the drug absorption in the small intestine and its absorption mechanisms (Hubatsch et al., 2007). Simultaneously, the active mechanisms of substances against UC can be explored by inducing a UC model in caco‐2 cells (Roselli et al., 2022). In vivo experiments with a colitis mouse model, besides detecting cell cytokines and tight junction (TJ) protein expression levels, researchers also analyze intestinal permeability and intestinal flora by RT‐qPCR and 16S rDNA sequencing technology (Wang et al., 2023).

Recently, soluble epoxide hydrolase (sEH, EC 3.3.2.10) has been selected as a target for curing diversity diseases as its function is to convert functional epoxyeicosatrienoic acids (EETs) to less/no active dihydroxyeicosatrienoic acids (DHETs), which will influence various pathological responses such as inflammation, hypertension, and neurodegeneration. Wang et al. (2018) indicated that sEH inhibitors can alleviate obesity‐induced inflammation of the colon. Meanwhile, deficiency or inhibition of sEH has long been shown to alleviate IBD in mice (Zhang et al., 2013). Therefore, sEH may be a novel therapeutic target for colitis, and finding colitis regulatory peptides by screening sEH inhibitors is a promising strategy. Notably, several food‐derived bioactive peptides have been reported to possess sEH inhibitory activity, which indicates that identifying colitis regulatory peptides by screening the inhibitory peptides of sEH is considered a promising approach for the treatment of inflammation and related diseases (Yang, 2018).

Peptides have been shown to alleviate colitis by improving antioxidant capacity and systemic anti‐inflammatory capacity, including the upregulation of anti‐inflammatory cytokines and the downregulation of pro‐inflammatory cytokines, as well as increasing the level of TJ proteins to maintain the intestinal tract barrier and balance the intestinal flora. However, the active mechanism elaboration of colitis regulatory peptides is not comprehensive and lacks systemic properties. To further promote the identification and mechanism analysis of colitis regulatory peptides, the structure analysis of colitis regulatory peptides will contribute to laying the foundation for the future research on the development and identification of related colitis regulatory peptides.

## 3 THE STRUCTURAL PROPERTIES OF COLITIS REGULATORY PEPTIDES

The sequence and structure are significantly important for peptides' functions, such as length and amino acid residues composition. However, there is almost no study focusing on the structure properties of colitis regulatory peptides. Therefore, we summarized the structural properties of reported colitis regulatory peptides in this review. As shown in Table 1, a total of 38 peptides with colonic inflammation regulatory effects were collected by searching the Web of Science (accessed on 30 Mar 2024).

**FIGURE 2:**
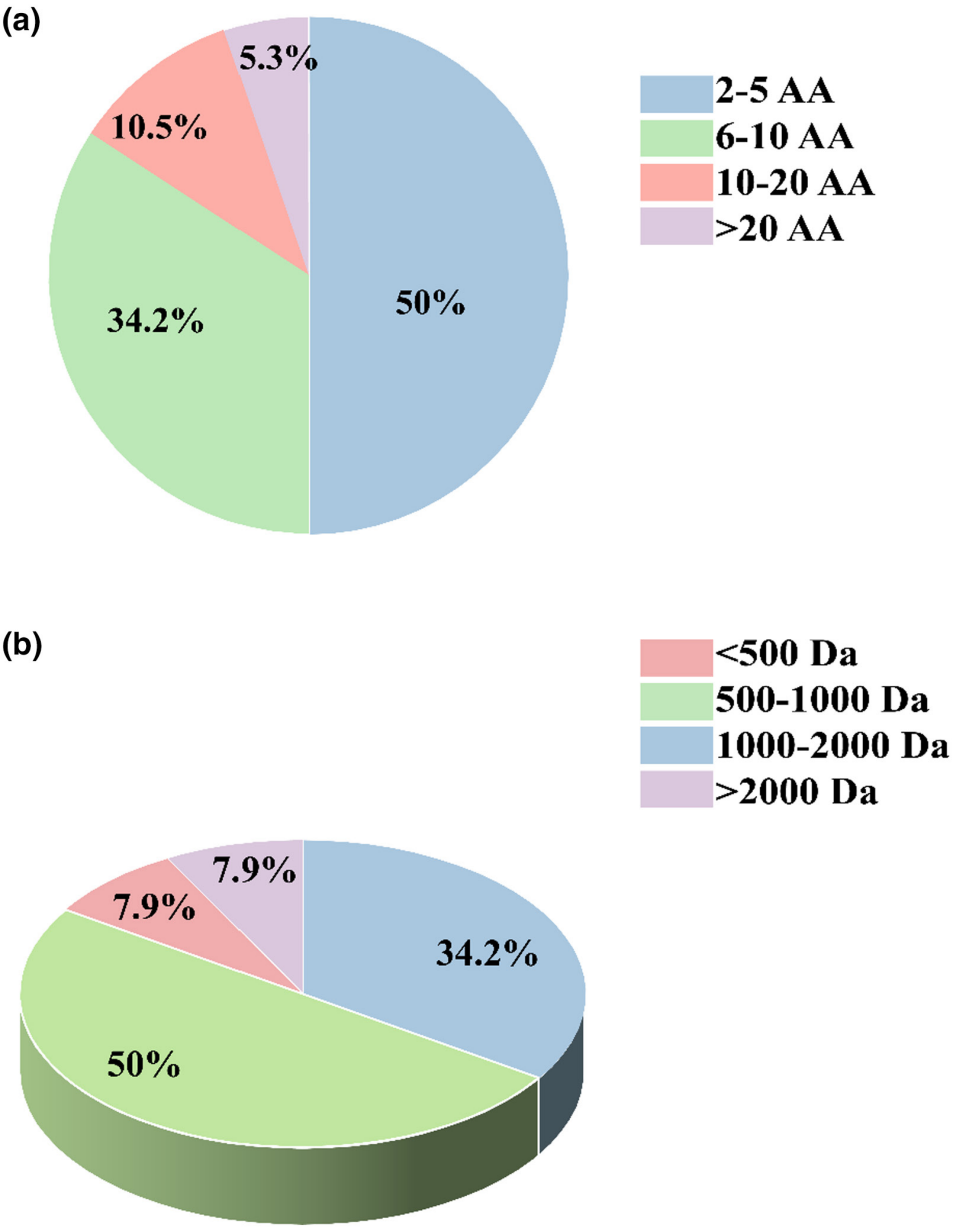
Characteristics of colitis regulatory peptides. (a) Length of peptide distribution; (b) molecular weight. The peptides with colitis regulatory activity were obtained from the Web of Science.

**FIGURE 3:**
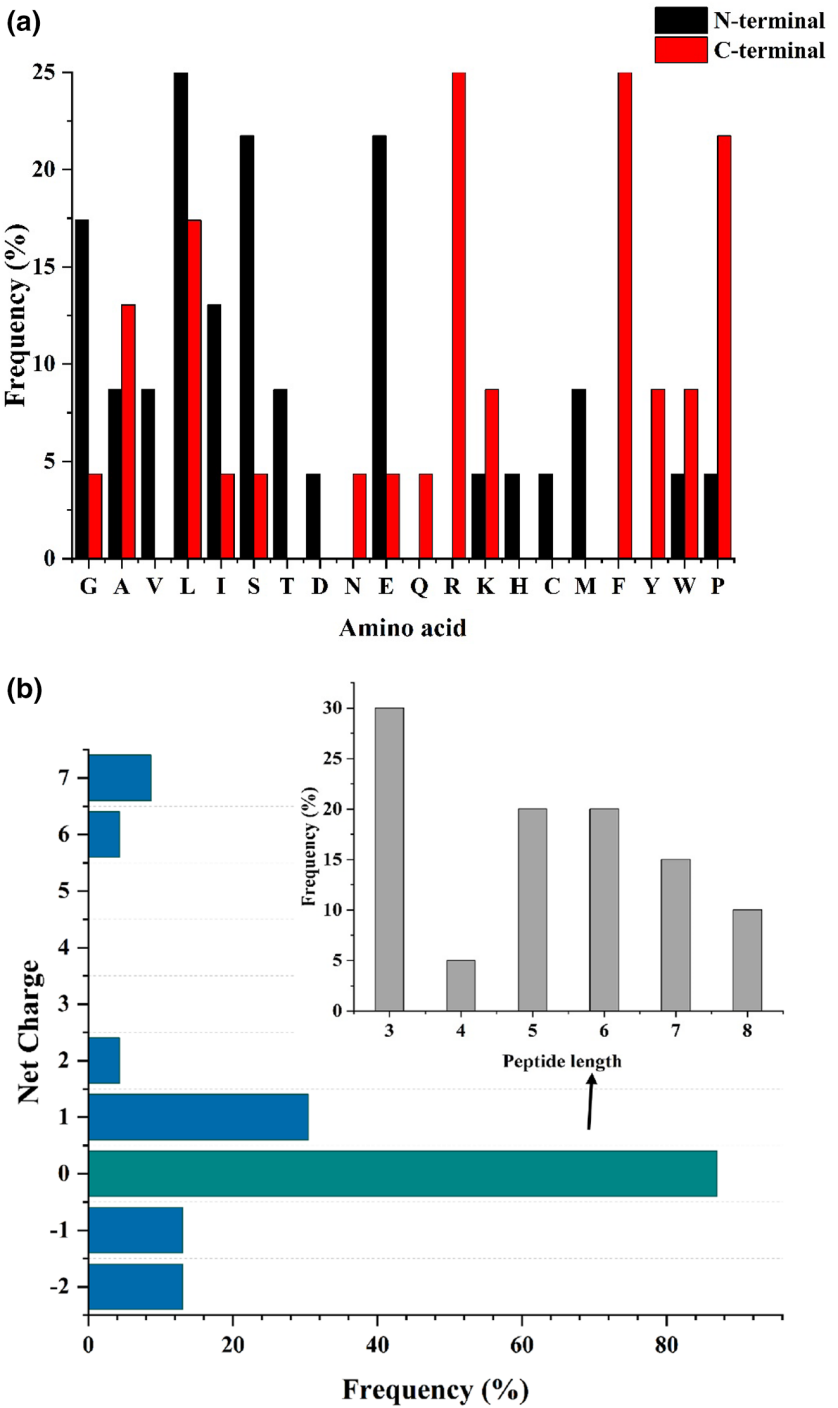
Amino acid compositions of the colitis regulatory peptides. (a) The frequency of each amino acid in the N‐terminal and C‐terminal of the colitis regulatory peptides. (b) The net charge distribution of colitis regulatory peptides and the peptide length distribution of colitis regulatory peptides with net charge of 0.

Normally, food‐derived bioactive peptides contain 2–20 amino acid residues (Chai et al., 2020). Studies showed that peptides' bioactivities and functions may be similar or the same if they have similar amino acid sequences (Tu et al., 2018). Hence, we analyzed the characteristics (number of amino acid residues, molecular weight, N‐terminal amino acid, C‐terminal amino acid, and net charge) of the colitis regulatory peptides (Figures 2 and 3, and Table S1).

Results demonstrated that most of the colitis regulatory peptides belong to small molecular peptides, within 2–10 amino acids (84.2%), among which 50% of peptides with 2–5 amino acid residues and 34.2% of peptides with 6–10 amino acid residues; peptides with >20 amino acid residues are rare (only 5.3%). In terms of molecular weight, it is found that most of the colitis regulatory peptides have a molecular weight between 500 and 2000 Da (84.2%), about 50% of the colitis regulatory peptides are with molecular weight <500–1000 Da, and 34.2% of those have a molecular weight between 1000 and 2000 Da. Saadi et al. (2015) pointed out that the kind of N‐terminal and C‐terminal amino acids would influence the activity of the peptide, and they found Phe (F), Tyr (Y), and Pro (P) are three residues that contribute most to the immune response activity. We statistically analyzed the N‐terminal and C‐terminal amino acids of colitis regulatory peptides, respectively. The most common N‐terminal amino acids are Leu (L), Ser (S), Glu (E), and Gly (G), while Arg (R) and Phe (F) are the most common C‐terminal amino acids, followed by Pro (P) and Leu (L). Additionally, we found most colitis‐regulating peptides studied by the researchers to be short peptides with a net charge of 0 (87%). We analyzed the net charge of the colitis regulatory peptides (Table S1), and about half of the peptides that have been found to have colitis regulatory activity are short peptides within 3–8 amino acids in length. Based on the present data, which may have several limitations as the number of peptides is not too large, we speculate that there may be several reasons for this phenomenon: peptide length and the net charge of peptides greatly affect their colitis anti‐inflammatory activity. Short peptides may include functional regions or biologically active parts of proteins that are likely to be associated with colitis regulatory activity, such as signaling peptides, domains, and active sites. Studies have shown that the transport, absorption, and utilization of small molecular weight peptides in vivo greatly affect the activity of peptides. Short peptides can be transported to intestinal cells by intestinal‐expressed peptide transporters, thus preserving their anti‐inflammatory activity fragments more completely (Karaś, 2019). Peptide length and degradation of peptides (and peptide sequences) by intestinal proteases may be factors determining their bioavailability (Shen & Matsui, 2017). Short peptides are more easily absorbed by intestinal transport and can avoid digestion by enzymes through intestinal transporters; therefore, they can make full use of their bioavailability and bioaccessibility and play a role in the body.

In conclusion, the colitis regulatory peptide activity is probably related to its N‐terminal and C‐terminal amino acids, molecular weight, length, and net charge. However, more deep analyses are needed in the future after more colitis regulatory peptides are identified.

## 4 ACTIVITY MECHANISMS OF THE COLITIS REGULATORY PEPTIDES

Nowadays, many studies about the food‐derived bioactive peptides regulating colitis have been conducted and found that they could alleviate colitis symptoms through multiple mechanisms. For example, tuna peptide was reported to have the function of alleviating colitis by strengthening the antioxidant and anti‐inflammatory capabilities as well as improving the intestinal tract barrier and metabolic disorder and regulating the intestinal flora (Xiang, Zhou, et al., 2021). Fish‐scale collagen peptide showed anti‐inflammatory effects on colitis mice by reducing myeloperoxidase (MPO)‐dependent activation of inflammatory cells, which can also inhibit the activation of the nuclear factor NF‐κB pathway and serum active monocyte chemotactic protein 1 (MCP‐1) (Azuma et al., 2014). Glutamine peptide can improve the antioxidant capacity, reduce the expression of inflammatory cytokines, decrease intestinal mucosa permeability, and protect the intestinal barrier of colitis mice (Jing et al., 2022). Zhou et al. (2012) confirmed that glutamine and arginine may have a synergistic effect on regulating colitis by reducing cell inflammatory cytokines expression; their mechanism is that arginine can promote the speed of glutamine synthesis. Here, we summarized the related research advancements of food‐derived colitis regulatory peptides, as well as discussed and divided their mechanisms into the following 5 parts.

### 4.1 Modulating intestinal microbiota

There are about 100 trillion microorganisms in the human gastrointestinal tract (Valdes et al., 2018), which mainly exist in the colon, including *Bacteroides*, *Bifidobacterium*, *Clostridium*, etc. Intestinal flora plays an important role in the maintenance of human health and microecological balance in the body, and its alteration can induce many acute and chronic diseases, such as cardiovascular disease (Jones & Neish, 2021; Kazemian et al., 2020), liver and kidney disease (Jones & Neish, 2021; Plata et al., 2019), and pulmonary disease (Zhang et al., 2020). The balance of intestinal flora is equally important in colitis patients and is a vital index for evaluating their disease extent.

*Firmicutes* and *Bacteroidetes* are two dominant bacterial phyla in the colon, whose populations can be quantified through high‐throughput 16S rDNA sequencing. Most healthy adults' intestinal microbiota is controlled by just two bacterial phyla: Gram‐positive *Firmicutes* and Gram‐negative *Bacteroides*, which account for more than 90% of the intestinal flora, followed by *Proteobacteria*, *Patescibacteria*, and *Actinobacteria* (Stojanov et al., 2020). Therefore, the ratio of *Firmicutes* to *Bacteroidetes* (F/B ratio) is frequently utilized to assess alterations in the gut microbiome. An elevated or decreased F/B ratio is considered dysecological, and colitis patients are often accompanied by a decreased F/B ratio (Kiyono et al., 2016; Yang et al., 2020; Zhou et al., 2021).

Food‐derived peptides can enrich potentially beneficial bacteria, reduce pathogenic bacteria, as well as increase the abundance of intestinal flora to improve UC. Xiang, Jiang, et al. (2021) found that shrimp peptide could increase the abundance and diversity of intestinal flora, and increase beneficial bacteria such as *Proteobacteria*, *Patescibacteria*, *Tenericutes*, and *Actinobacteria*. Li et al. (2022) found that co‐treatment with tetrapeptide from maize and probiotics could exert a strong anti‐inflammatory effect through modulating gut microbiota and normalizing the ratio of F/B. Peptide LPF, derived from walnut protein, could improve the ratio of F/B in colitis mice, increase the abundance of beneficial genera such as the family of *Lachnospiraceae* and *Ruminococcaceae*, and reduce the harmful genera such as *Bilophila* (Zhi et al., 2022). Cai et al. (2019) also found that the microbiota composition in colitis mice could be reversed by increasing the potential beneficial gut bacteria and decreasing the harmful gut bacteria. Ge et al. (2021) confirmed that egg white peptides could achieve an immune‐modulating effect by regulating the gut microbiota and also inhibiting the expression of pro‐inflammatory cytokines. In addition, the intestinal flora imbalance will also strongly affect the integrity of the intestinal mucosa and intestinal permeability, thus exacerbating the colonic inflammation (Guo et al., 2020).

### 4.2 Decreasing intestinal epithelial permeability

**FIGURE 4:**
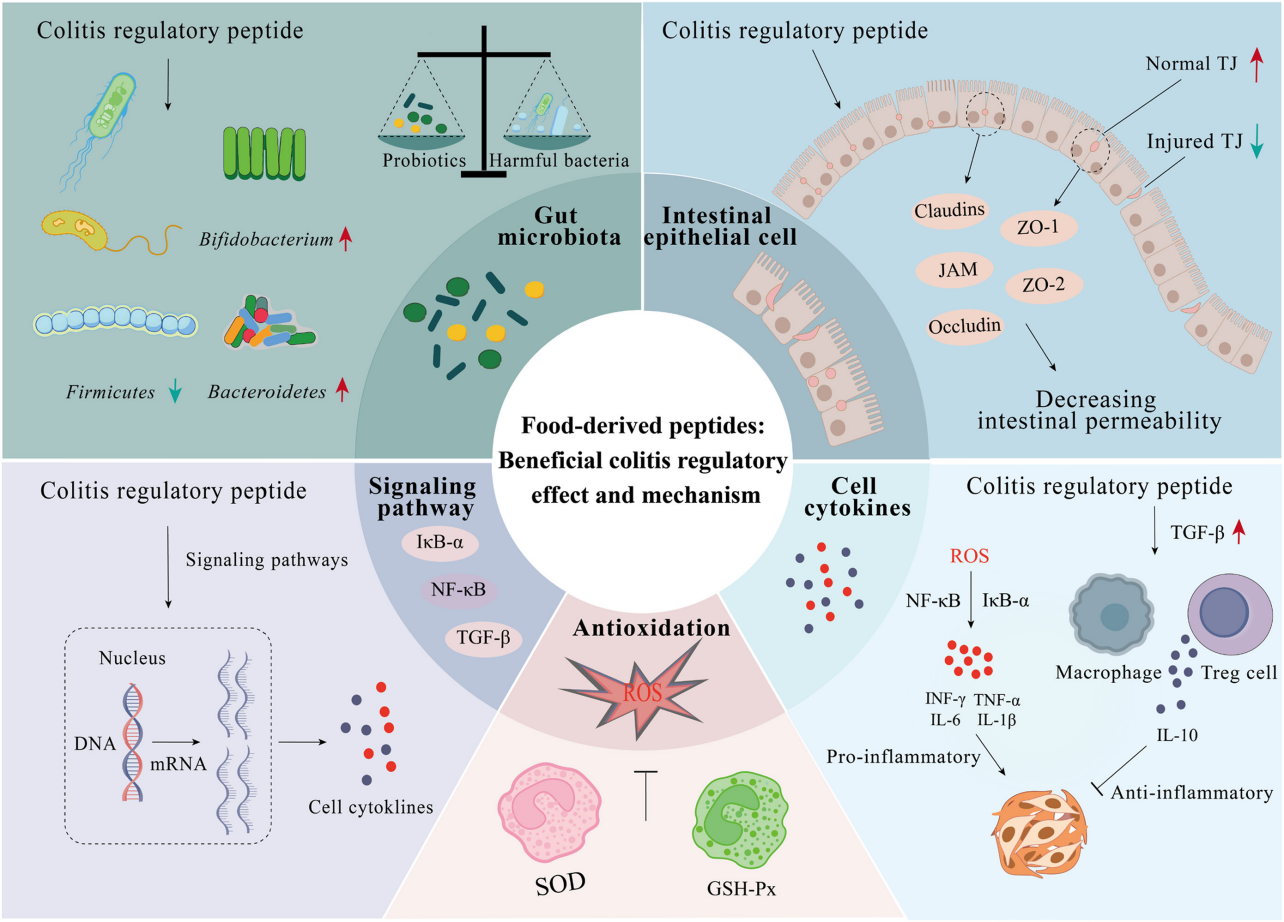
Mechanisms of colitis regulatory peptides. The mechanisms include modulating gut microbiota balance; decreasing intestinal epithelium permeability; increasing antioxidant ability; regulating inflammatory cytokine expression; and targeting signaling pathways.

It is a normal phenomenon to see the damaged intestine structure and increased intestinal epithelial permeability in colitis mice (Figure 4). Tight junction proteins are the apical junctional complex between epithelial and endothelial cells, which is one of the determinants of epithelial permeability (Li, Ge, et al., 2021), including TJ protein‐1 (Zonula occludens‐1, ZO‐1), claudin‐1 (Cldn‐1) (Song et al., 2019), junctional adhesion molecule (JAM), etc. Occludin is the main TJ protein construction between cells, which maintains cell polarity and TJ barrier function. DSS induced a decrease in TJ protein expression in colitis mouse colon tissues (Zhang et al., 2023). Studies showed that antarctic krill oil could significantly improve the mRNA expression of claudin‐1, occludin, and ZO‐1 in the DSS‐induced UC mice colon tissue and thus decrease the intestinal permeability (Zhou et al., 2021). *Tricholoma matsutake* mushroom peptide could regulate the TJ protein to enhance the intestinal barrier by detecting the change of ZO‐1 and occludin in colitis mice (Li, Ge, et al., 2021).

Some enzymes and signaling pathways were confirmed to be associated with intestinal barrier integrity, such as Src kinase and aryl hydrocarbon receptor (AhR) (Li et al., 2020). Diamine oxidase (DAO) and lipopolysaccharide (LPS) are the symbolic substances of the intestinal mucosa and will reflect changes in intestinal permeability. DAO exists in human and other mammalian intestinal mucosa and is a highly reactive cytoplasmic enzyme in the upper layer of mammalian small intestinal villi. When intestinal mucosal epithelial cells are damaged, intracellular DAO is released into the intestines and blood, increasing the blood DAO level and intestinal permeability. DAO and LPS levels are high in colitis. Several studies have confirmed that shrimp peptide (Xiang, Jiang, et al., 2021), antarctic oil krill (Zhou et al., 2021), and corn protein hydrolysates with glutamine‐rich peptides (Yang et al., 2020) are effective in alleviating colitis symptoms by detecting a decrease in serum DAO and LPS levels. The level of serum FITC‐glucan is also used to assess intestinal permeability. *Tricholoma matsutake* peptides can reduce the level of serum FITC‐glucan and the content of DAO in serum and regulate intestinal permeability (Li, Ge, et al., 2021).

### 4.3 Increasing antioxidant ability

Superoxide dismutase (SOD) and glutathione peroxidase (GSH‐Px) are the most common enzymes related to oxidative stress in the mouse colitis model. The inflammatory response in the body of mice can be effectively judged by detecting the contents of SOD and GSH‐Px in the serum and tissues of mice, thus reflecting the effectiveness of the studied substances for colitis. Shrimp peptide (Xiang, Jiang, et al., 2021) could alleviate the oxidative stress of DSS‐induced UC mice by improving the serum SOD and GSH‐Px levels. Starting with antioxidants may sprout out new treatments for colitis, and some carrier delivery systems or encapsulated delivery applications, such as astaxanthin nanoparticles, have significantly improved the bioaccessibility of the active substance (Zhang et al., 2023).

Nitric oxide synthase (NOS) belongs to the nervous system, which has three subtypes: nNOS, eNOS, and iNOS. iNOS, a rate‐limiting enzyme of NO that synthesizes and induces the secretion of a large amount of NO, induces body oxidative stress and stimulates inflammation (Zhang et al., 2015). DSS‐induced colitis leads to significant increases in iNOS and cyclooxygenase (COX‐2), and iNOS may be directly regulated by the c‐jun signaling pathway (Hu et al., 2020).

### 4.4 Regulating the expression of inflammatory cytokines

Inflammatory cytokines are involved in human body inflammation, mainly including anti‐inflammatory cytokines (IL‐10) and pro‐inflammatory cytokines (TNF‐α, IL‐1β, IL‐6, IL‐8, etc.), which can activate the signaling pathways and induce the production of prostaglandin synthetase like COX‐2 and NOS. All of these are the leading indexes to assess DSS‐induced colitis in mice. Xing et al. (2022) conducted in vivo experiments in mice with active peptides produced during the fermentation of dry‐cured Xuanwei ham and found that they ameliorated DSS‐induced colitis symptoms by regulating the secretion of TNF‐α, IL‐6, and MCP‐1. Wang et al. (2019) found *Katsuwonus pelamis* enzymatic peptide (SEP) could evidently reduce the expression of IL‐6 and TNF‐α and increase the expression of IL‐10, which confirmed SEP has anti‐UC activity. Singh et al. (2022) found that Saffron treatment could decrease the pro‐inflammatory macrophages and increase the anti‐inflammatory macrophages, which influenced the inflammatory cytokine expression of TNF‐α and IL‐10. ELISA assay kits are usually used to detect the levels of IL‐1β, IL‐6, TNF‐α, IL‐10, and COX‐2 (Zhang et al., 2015) in the mouse serum. It is also possible to determine the amount of cytokines and TJ proteins by measuring the amount of some mRNAs by RT‐qPCR (Yi et al., 2020; Zhou et al., 2021).

Myeloperoxidase (MPO) is a functional and activation marker of neutrophils, and it is positively correlated with the degree of neutrophil infiltration in colon tissue, which often happens in the Lamina propria and submucosa of DSS‐induced colonic tissue (Li et al., 2013; Zhang et al., 2015). Besides, the colon of colitis mice was accompanied by numerous infiltrations of eosinophils (Li et al., 2020). Eosinophil peroxidase (EPO) was demonstrated to be a key disease mediator in UC and was found to manifest at high levels in UC patients (Forbes et al., 2004; Makiyama et al., 1995). DSS‐induced colitis resulted in higher levels of MPO and EPO in the intestine compared to healthy ones. Human fecal microbiota transplantation is verified to reduce the susceptibility to DSS‐induced colitis by decreasing the MPO/EPO and some other cell cytokine expressions (Yang, Zheng, et al., 2022).

### 4.5 Targeting signaling pathways

It has been studied that there are several signaling pathways correlated with colitis, involving NF‐κB, MAPK, TGF‐β1, and IκB‐α, etc. Zhang et al. (2015) found that the peptide cathelicidin‐BF could alleviate the DSS‐induced inflammation by inhibiting the NF‐κB pathway, whose activation might be influenced by the phosphorylation level of IκB‐α and p65 (Zhou et al., 2021); what is more, it could induce a decrease of peroxisome proliferator‐activated receptor gamma (PPAR‐γ) in UC (Dubuquoy et al., 2003). Ahmedy et al. (2020) confirmed that melittin has an alleviation effect on colitis by regulating NF‐κB and p38MAPK pathways, and also decreases the level of COX‐2 in colon tissue. Zheng et al. (2016) confirmed that a peptide from *Hydrophis cyanocinctus* could attenuate DSS‐induced colitis by inhibiting TNF‐α‐mediated activation of NF‐κB and MAPK pro‐inflammatory signaling pathways. Milk‐derived casein glycomacropeptide has an anti‐inflammatory effect on UC mice through the NF‐κB/p65 and TGF‐β1 pathways (Chen et al., 2014). By the way, abnormality in TGF‐β is a mediator factor for the pathogenesis of IBD (Bai et al., 2022). Lee et al. (2016) found that TGF‐β‐deficient Treg cells were unable to regulate myeloid‐derived suppressor cells (MDSC) function in an experiment‐induced model of colitis, but the TGF‐β‐mediated in vitro‐differentiated MDSC experiments showed favorable colitis prevention. Deng et al. (2020) discovered that GPA could increase the expression of Nur77 in intestinal epithelial cells, which might be a block of NF‐κB activation, and could inhibit the upstream of the NF‐κB pathway through upregulating IκBα. Furthermore, some other signaling pathways have been mentioned by research; for example, Kelch‐like ECH‐associating protein 1 (Keap1)‐nuclear factor E2‐related factor 2 (Nrf2) signaling pathway was confirmed to be a predominant factor in the study of DSS‐induced colitis by Yang, Huang, et al. (2022),  which would be activated by rice protein peptides. Besides, these peptides also increased the F/B and the beneficial bacteria (*Akkermansia*), as well as the short‐chain fatty acids level (Yang, Huang, et al., 2022). Li et al. (2020) demonstrated that the pentapeptide of LNLYP could activate the aryl hydrocarbon receptor (AhR) signaling pathway, which also modulated the Src kinase so as to regulate intestinal epithelium permeability.

## 5 CONCLUSION AND FUTURE PERSPECTIVES

Currently, more people are suffering from IBD worldwide. It is becoming significant in preventing colitis and choosing safe and efficient natural compounds instead of drugs for patients with colitis. In vitro and in vivo studies showed that the mechanisms of food‐derived bioactive peptides against ulcerative colitis mainly include 5 aspects: modulating intestinal microbiota, decreasing intestinal epithelial permeability, increasing antioxidant ability, regulating the expression of inflammatory cytokines, and targeting signaling pathways. Recent studies showed that there are still some aspects that could be improved for the study and application of food bioactive peptides in alleviating colitis:

1. Identifying the sequences of peptides and analyzing their active mechanisms in regulating colitis require further study.
2. A more in‐depth study about the commonalities in structure characteristics, dose‐effect relationships, and structure‐effect relationships of peptides that can alleviate or prevent colitis symptoms needs to be conducted.
3. Seeking novel targets or further study of metabolic pathways can help discover novel bioactive peptides and analyze the action of mechanisms regulating colitis.
4. It is promising to develop more relevant products, such as foods, health care products, and drugs combined with carrier delivery systems or embedding applications, and improve their bioaccessibility.

In summary, a lot of research has been conducted on food‐derived bioactive peptides regulating colitis but lacks deep mechanism elaboration as well as structure–effect relation analysis. More novel food‐derived peptides are expected to be analyzed and developed in the future. More attention should be paid to the application of food‐derived peptides in regulating colitis.

## AUTHOR CONTRIBUTIONS

**Wenpei Qiu**: Writing – original draft (equal), Writing – review & editing. **Zhicheng Wang**: Writing – original draft (equal), Writing – review & editing. **Qirui Liu**: Data curation. **Qiwei Du**: Conceptualization. **Xiaoqun Zeng**: Supervision. **Zhen Wu**: Validation. **Daodong Pan**: Supervision. **Xiaohong Zhang**: Supervision, Funding acquisition. **Maolin Tu**: Funding acquisition, Supervision, Writing – review & editing.

## CONFLICT OF INTEREST STATEMENT

The authors declare no conflicts of interest.

## Supporting information


Table S1

## Data Availability

Data presented in this study are contained in the article.
