# Supplementary material for: Structure and regulatory mechanisms of food‐derived peptides in inflammatory bowel disease: A review
Source: Food Sci Nutr. 2024 Jun 18;12(9):6055–69. doi: 10.1002/fsn3.4228 (PMC11561845; doi:10.1002/fsn3.4228)
Supplement: Supplementary file 1 — Table S1 [file FSN3-12-6055-s001.docx]

**Supplementary material**

**Structure and regulatory mechanisms of food-derived peptides in inflammatory bowel disease: A review**

**Table S1** Molecular information of colitis regulatory peptides.

| No. | Peptide Sequence | Peptide Source | Sequence length | Molecular Weight | Net Charge | Reference |
| --- | --- | --- | --- | --- | --- | --- |
|  | pyroEY | Pyro-Glutamyl peptides | 2 | 310.30 | -1 | Kiyono et al. (2016) |
|  | pyroENI |  | 3 | 374.39 | -1 |  |
|  | pyroEL | Enzymatic hydrolysate of wheat gluten | 2 | 260.29 | -1 | Wada et al. (2013) |
|  | LPF | Walnut protein | 3 | 375.46 | 0 | Zhi et al. (2022) |
|  | VPY | Soy | 3 | 377.43 | 0 | Kovacs-Nolan et al. (2012) |
|  | IRW | Egg protein transferrin | 3 | 473.57 | 1 | Liu et al. (2018) |
|  | IQW |  | 3 | 445.51 | 0 |  |
|  | GPA | Fish skin gelatin | 3 | 243.26 | 0 | Deng, Zheng, et al. (2020) |
|  | GPR | Fish skin gelatin | 3 | 328.37 | 1 | Deng, Cui, et al. (2020) |
|  | GP(Hyp) |  | 3 | 285.30 | 0 |  |
|  | L(Hyp)G |  | 3 | 301.34 | 0 |  |
|  | (Hyp)P |  | 2 | 228.24 | NaN |  |
|  | IPVA | Whey protein | 4 | 398.50 | 0 | Oyama et al. (2017) |
|  | LNLYP | Chicken by-product | 5 | 618.72 | 0 | Li et al. (2020) |
|  | LPLLR | Walnuts | 5 | 610.79 | 1 | Qi et al. (2023) |
|  | SHTLP |  | 5 | 553.61 | 0 | Chen et al. (2023) |
|  | HYNLN |  | 5 | 659.69 | 0 |  |
|  | LGTYP |  | 5 | 549.62 | 0 |  |
|  | GLTSK | *Bean* | 5 | 504.58 | 1 | Luna-Vital et al. (2017) |
|  | EAMAPK | *Stracchino* cheese | 6 | 645.77 | 0 | Pepe et al. (2016) |
|  | AVPYPQ |  | 6 | 673.76 | 0 |  |
|  | TPGAFF | Quinoa | 6 | 638.71 | 0 | Wang et al. (2024) |
|  | VSAAAA | Millet gliadin | 6 | 488.54 | 0 | Hong et al. (2023) |
|  | MLGATSL | Preserved duck egg white | 7 | 691.84 | 0 | Zhang et al. (2018) |
|  | SLSFASR |  | 7 | 766.84 | 1 |  |
|  | MSYSAGF |  | 7 | 761.84 | 0 |  |
|  | DEDTQAMPFR |  | 10 | 1209.29 | -2 |  |
|  | LLTRAGL | Rapana venosa | 7 | 742.91 | 1 | Cao et al. (2024) |
|  | SSEDIKE | Amaranth protein | 7 | 806.82 | -2 | Fernandez-Tome et al. (2019) |
|  | WFNNAGP | *Tricholoma matsutake* | 7 | 804.85 | 0 | Li, Lv, et al. (2021) |
|  | SDIKHFPF | *Tricholoma matsutake Singer* | 8 | 990.11 | 0 | Li, Ge, et al. (2021) |
|  | SDLKHFPF |  | 8 | 990.11 | 0 |  |
|  | APEPEPAF | Wheat germ-derived peptide | 8 | 856.92 | -2 | Wang et al. (2023) |
|  | CKYVCTCKMS | *Buffalo Mozzarella* Cheese | 10 | 1165.48 | 2 | Tenore et al. (2019) |
|  | PVLGPVRGPFPLL | Fresh wheat germ and apple | 13 | 1361.67 | 1 | He et al. (2022) |
|  | KCRQWQSKIRRTNPIFCIRR | Porcine lactoferrin | 20 | 2590.09 | 7 | Zong et al. (2016) |
|  | TK(C)FQWQRNMRKVRGPPVS(C)IKR | Human lactoferrin | 23 | 2637.12 | 7 | Håversen et al. (2003) |
|  | EATKCFQWQRNMRKVRGPPVSCIKR |  | 25 | 3019.58 | 6 |  |

**References**

Cao, Y., Xu, F., Xia, Q., Liu, K., Lin, H., Zhang, S., & Zhang, Y. (2024). The peptide LLTRAGL derived from rapana venosa exerts protective effect against inflammatory bowel disease in zebrafish model by regulating multi-pathways. *Marine Drugs, 22*(3), 100.

Chen, Z., Zhang, Y., Zhang, X., Li, X., Ma, J., Jing, X., & Wang, X. (2023). Anti-inflammatory effect of a novel millet gliadin peptide on mice with colitis. *Journal of Functional Foods, 111*, 105912.

Deng, Z., Cui, C., Wang, Y., Ni, J., Zheng, L., Wei, H.-K., & Peng, J. (2020). FSGHF3 and peptides, prepared from fish skin gelatin, exert a protective effect on DSS-induced colitis via the Nrf2 pathway. *Food & Function, 11*(1), 414-423. <http://doi.org/10.1039/c9fo02165e>

Deng, Z., Zheng, L., Xie, X., Wei, H., & Peng, J. (2020). GPA peptide enhances Nur77 expression in intestinal epithelial cells to exert a protective effect against DSS‐induced colitis. *The FASEB Journal, 34*(11), 15364-15378. <http://doi.org/10.1096/fj.202000391RR>

Fernandez-Tome, S., Hernandez-Ledesma, B., Chaparro, M., Indiano-Romacho, P., Bernardo, D., & Gisbert, J. P. (2019). Role of food proteins and bioactive peptides in inflammatory bowel disease. *Trends in Food Science & Technology, 88*, 194-206.

Håversen, L., Baltzer, L., Dolphin, G., Hanson, L., & Mattsby‐Baltzer, I. (2003). Anti‐inflammatory activities of human lactoferrin in acute dextran sulphate‐induced colitis in mice. *Scandinavian Journal of Immunology, 57*, undefined.

He, D., Zeng, W., Wang, Y., Xing, Y., Xiong, K., Su, N., Zhang, C., Lu, Y., & Xing, X. (2022). Isolation and characterization of novel peptides from fermented products of Lactobacillus for ulcerative colitis prevention and treatment. *Food Science and Human Wellness, 11*(6), 1464-1474. <http://doi.org/10.1016/j.fshw.2022.06.003>

Hong, Z., Shi, C., Hu, X., Chen, J., Li, T., Zhang, L., Bai, Y., Dai, J., Sheng, J., & Xie, J. (2023). Walnut protein peptides ameliorate DSS-induced ulcerative colitis damage in mice: an in silico analysis and in vivo investigation. *Journal of Agricultural and Food Chemistry, 71*(42), 15604-15619.

Kiyono, T., Wada, S., Ohta, R., Wada, E., Takagi, T., Naito, Y., Yoshikawa, T., & Sato, K. (2016). Identification of pyroglutamyl peptides with anti-colitic activity in Japanese rice wine, sake, by oral administration in a mouse model. *Journal of Functional Foods, 27*, 612-621. <http://doi.org/10.1016/j.jff.2016.10.014>

Kovacs-Nolan, J., Zhang, H., Ibuki, M., Nakamori, T., Yoshiura, K., Turner, P. V., Matsui, T., & Mine, Y. (2012). The PepT1-transportable soy tripeptide VPY reduces intestinal inflammation. *Biochimica et Biophysica Acta (BBA)-General Subjects, 1820*(11), 1753-1763. <http://doi.org/10.1016/j.bbagen.2012.07.007>

Li, M., Ge, Q., Du, H., Jiang, P., Bao, Z., Chen, D., & Lin, S. (2021). Potential mechanisms mediating the protective effects of Tricholoma matsutake-derived peptides in mitigating DSS-induced colitis. *Journal of Agricultural and Food Chemistry, 69*(19), 5536-5546. <http://doi.org/10.1021/acs.jafc.1c01908>

Li, M., Lv, R., Wang, C., Ge, Q., Du, H., & Lin, S. (2021). Tricholoma matsutake-derived peptide WFNNAGP protects against DSS-induced colitis by ameliorating oxidative stress and intestinal barrier dysfunction. *Food & Function, 12*(23), 11883-11897.

Li, S., Ma, B., Wang, J., Peng, H., Zheng, M., Dai, W., & Liu, J. (2020). Novel pentapeptide derived from chicken by-product ameliorates DSS-induced colitis by enhancing intestinal barrier function via AhR-induced Src inactivation. *Journal of Agricultural and Food Chemistry, 68*(48), 14192-14203. <http://doi.org/10.1021/acs.jafc.0c06319>

Liu, G., Yan, W., Ding, S., Jiang, H., Ma, Y., Wang, H., & Fang, J. (2018). Effects of IRW and IQW on oxidative stress and gut microbiota in dextran sodium sulfate-induced colitis. *Cellular Physiology and Biochemistry, 51*(1), 441-451. <http://doi.org/10.1159/000495240>

Luna-Vital, D. A., González de Mejía, E., & Loarca-Piña, G. (2017). Dietary Peptides from Phaseolus vulgaris L. Reduced AOM/DSS-Induced Colitis-Associated Colon Carcinogenesis in Balb/c Mice. *Plant Foods for Human Nutrition*. <http://doi.org/10.1007/s11130-017-0633-2>

Oyama, M., Van Hung, T., Yoda, K., He, F., & Suzuki, T. (2017). A novel whey tetrapeptide IPAV reduces interleukin-8 production induced by TNF-α in human intestinal Caco-2 cells. *Journal of Functional Foods, 35*, 376-383. <http://doi.org/10.1016/j.jff.2017.06.001>

Pepe, G., Sommella, E., Ventre, G., Scala, M. C., Adesso, S., Ostacolo, C., Marzocco, S., Novellino, E., & Campiglia, P. (2016). Antioxidant peptides released from gastrointestinal digestion of "Stracchino" soft cheese: Characterization, in vitro intestinal protection and bioavailability. *Journal of Functional Foods, 26*, 494-505. <http://doi.org/10.1016/j.jff.2016.08.021>

Qi, Y., Wu, D., Fang, L., Leng, Y., Wang, X., Liu, C., Liu, X., Wang, J., & Min, W. (2023). Anti-inflammatory effect of walnut-derived peptide via the activation of Nrf2/Keap1 pathway against oxidative stress. *Journal of Functional Foods, 110*, 105839.

Tenore, G. C., Pagano, E., Lama, S., Vanacore, D., Di Maro, S., Maisto, M., Capasso, R., Merlino, F., Borrelli, F., & Stiuso, P. (2019). Intestinal anti-inflammatory effect of a peptide derived from gastrointestinal digestion of buffalo (Bubalus bubalis) mozzarella cheese. *Nutrients, 11*(3), 610. <http://doi.org/10.3390/nu11030610>

Wada, S., Sato, K., Ohta, R., Wada, E., Bou, Y., Fujiwara, M., Kiyono, T., Park, E. Y., Aoi, W., & Takagi, T. (2013). Ingestion of low dose pyroglutamyl leucine improves dextran sulfate sodium-induced colitis and intestinal microbiota in mice. *Journal of Agricultural and Food Chemistry, 61*(37), 8807-8813. <http://doi.org/10.1021/jf402515a>

Wang, F., Chen, Y., Itagaki, K., Zhu, B., Lin, Y., Song, H., Wang, L., Xiong, L., Weng, Z., & Shen, X. (2023). Wheat Germ-Derived Peptide Alleviates Dextran Sulfate Sodium-Induced Colitis in Mice. *Journal of Agricultural and Food Chemistry, 71*(42), 15593-15603.

Zhang, M., Zhao, Y., Wu, N., Yao, Y., Xu, M., Du, H., & Tu, Y. (2018). The anti-inflammatory activity of peptides from simulated gastrointestinal digestion of preserved egg white in DSS-induced mouse colitis. *Food Funct, 9*(12), 6444-6454. <http://doi.org/10.1039/c8fo01939h>

Zhi, T., Hong, D., Zhang, Z., Li, S., Xia, J., Wang, C., Wu, Y., Jia, Y., & Ma, A. (2022). Anti-inflammatory and gut microbiota regulatory effects of walnut protein derived peptide LPF in vivo. *Food Res Int, 152*, 110875. <http://doi.org/10.1016/j.foodres.2021.110875>

Zong, X., Hu, W., Song, D., Li, Z., Du, H., Lu, Z., & Wang, Y. (2016). Porcine lactoferrin-derived peptide LFP-20 protects intestinal barrier by maintaining tight junction complex and modulating inflammatory response. *BIOCHEMICAL PHARMACOLOGY, 104*, 74-82. <http://doi.org/10.1016/j.bcp.2016.01.009>
